# Visual conflict and cognitive load modify postural responses to vibrotactile noise

**DOI:** 10.1186/1743-0003-11-6

**Published:** 2014-01-13

**Authors:** Emily A Keshner, Jill C Slaboda, Lois Lanaria Day, Kurosh Darvish

**Affiliations:** 1 Department of Physical Therapy, Temple University, 3307 N. Broad St., 19140 Philadelphia, PA, USA; 2 Department of Electrical and Computer Engineering, Temple University, 19122 Philadelphia, PA, USA; 3 Department of Mechanical Engineering, Temple University, 19122 Philadelphia, PA, USA

**Keywords:** *Balance*, *Virtual reality*, *Stochastic resonance*, *Approximate entropy*, *Attention*

## Abstract

**Background:**

Underlying the increased incidence of falls during multitasking is a reduced ability to detect or attend to the sensory information signaling postural instability. Adding noise to a biological system has been shown to enhance the detection and transmission of weakened or sub-threshold cutaneous signals. If stochastic resonance is to become an effective adjunct to rehabilitation, we need to determine whether vibrotactile noise can be effective when added to an environment presenting with other sensory noise.

**Methods:**

Sub-threshold vibration noise was applied for 30 sec at the soles of the feet in 21 healthy adults (20–29 yrs) between two 30-sec periods of no vibration. During the trials, subjects stood quietly with eyes closed or while viewing a visual scene that rotated in continuous upward pitch at 30 deg/sec. Subjects were also tested with these two visual conditions while performing a mental calculation task. It was hypothesized that sub-threshold vibration would increase regularity of postural sway, thereby improving postural stabilization during an attention demanding task but exerting less effect with multiple sensory demands. An ellipse fit to the covariance matrix revealed excursion of center of pressure (COP) and center of mass (COM) responses in the anterior-posterior and lateral planes. RMS values and approximate entropy of the COP and COM were calculated and statistically compared.

**Results:**

The addition of vibrotactile noise to the plantar surface during quiet stance with eyes closed reduced the area of the COM and COP responses, which then returned to pre-vibration levels after vibration was removed. Postural sway was generally increased with both visual field rotations and mental calculation compared to the eyes closed condition. The effect of sub-threshold vibratory noise on postural behavior was modified when visual field rotations and mental calculation was combined. It was shown that the measure of approximate entropy reflected increased task complexity.

**Conclusions:**

Our results suggest that the impact of destabilizing signals is modulated when combined with vibrotactile stimulation. The strong aftereffects of the vibration stimulus suggest that the system has adapted to the sensory array even in the short time period tested here. The results imply that application of vibrotactile stimulation has the potential for diminishing sway magnitudes while increasing the potential for response variability, thereby presenting a non-invasive method of reducing the potential for falls.

## Introduction

Our daily actions require that we maintain balance while attending to and performing a variety of other cognitive and motor tasks. Performing attention demanding tasks, however, has been shown to interfere with postural control [1,2]. In particular, aging individuals and those with balance problems are sensitive to this interference, which could boost the potential for fall related injury and reduced function in these populations [3].

A reduced ability to detect or attend to the sensory information signaling postural instability might underlie the increased incidence of falls during multitasking. For example, vibrotactile detection thresholds increase with age [4], thereby impairing the ability to detect changes in upright position and increasing the incidence of falls [5]. However, adding noise to a biological system has been shown to enhance the detection and transmission of weakened or sub-threshold cutaneous signals [6,7]. This phenomenon of stochastic resonance has been observed as a response from a nonlinear system to weak periodic [8] and aperiodic [9] input that is optimized by the presence of a nonzero level of noise. Using stochastic resonance, the ability to detect a weak broadband stimulus was augmented in mammalian cutaneous mechanoreceptors [6] and activation of somatosensory cortex and thalamus was observed through fMRI during vibrotactile stimulation [10,11].

In this study, we examined the effect of sub-threshold vibrotactile noise on the ability to maintain balance in healthy young adults while processing conflicting sensory feedback and performing a cognitively demanding task. We chose to study this population to determine whether vibrotactile noise has a meaningful effect on normal postural behavior when added in an environment presenting with other demands such as sensory conflict and additional cognitive load. Healthy young adults stood quietly on a compliant foam surface that has been shown to increase the postural sway response because of decreased reliability of plantar and proprioceptive feedback at the foot [12]. Postural instability was then modified either by removing vision by closing the eyes, or adding the destabilizing effects of visual field rotation [13,14]. Finally, cognitive demands were increased with the additional requirement of a mental calculation task. Sub-threshold vibration was applied after a period of accommodation to the visual motion, and was removed following the same period of time to determine the presence of after-effects. We hypothesized that sub-threshold vibration would increase regularity of postural sway and would thereby improve postural stabilization when attention was diverted but would be less effective when processing multiple sensory demands.

## Methods

### Subjects

Twenty-one healthy adults (20–29 yrs) gave informed consent to participate as approved by the Institutional Review Board at Temple University. All subjects had a minimum of 20/40 corrected vision in each eye and no history of central or peripheral neurological disorders or problems related to movements of the spinal column (e.g., significant arthritis or musculoskeletal abnormalities). Subjects reported intact light touch to a 10-g Semmes-Weinstein Monofilament placed on the great toe and lateral aspect of both feet. Vibrotactile sensitivity was reported as present to application of a 128 Hz tuning fork on the heel of each foot.

### Vibrotactile apparatus

**Figure 1:**
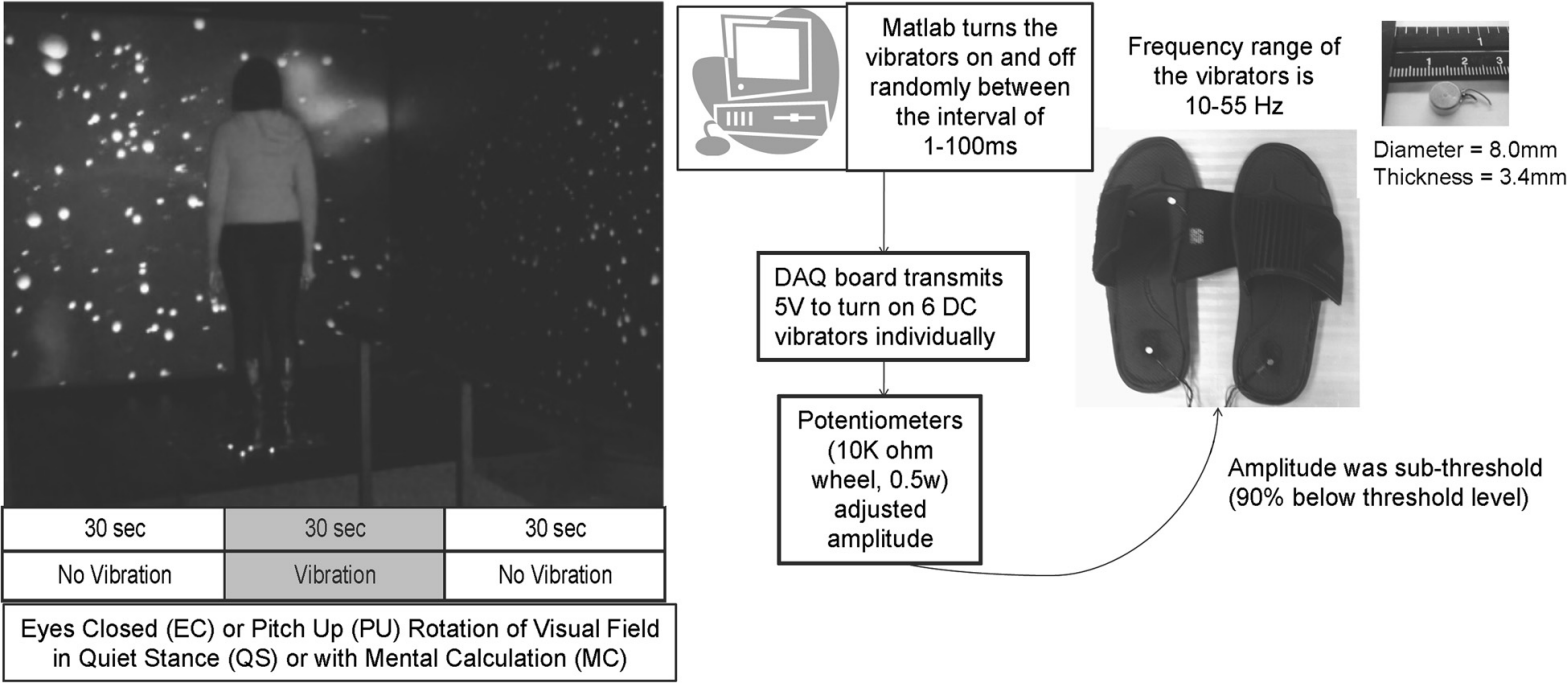
**Equipment and protocol used in this study.** (*Left*) An image of a subject standing before the visual scene projected within the virtual environment. Beneath is a diagram of the protocol for the eyes closed and constant upward pitch rotation of the visual field conditions. The vibration stimulus was sub-threshold for each subject. For each condition, subjects were either standing quietly or performing a mental calculation task. The first 30 seconds and the last 30 seconds of the trial had no vibration. Vibration was turned on during the middle 30 seconds of each trial. (*Right*) Diagram of the vibrating flip-flops showing the connection between the vibrators and the computer for data collection. Image shows the scale of the DC Vibrator Motor in cm.

Three vibrating elements, coin-shaped DC vibrating motors (diameter 8.0 ± 0.05 mm, 10–55 Hz and 3.40 mm thick) were embedded in a pair of flip-flops (Figure 1, right). Two were placed at the distal end of the first and fourth metatarsals and one at each heel in order to be sure vibration was received at the primary weight bearing and innervation areas of each foot [15]. Each element received a signal that turned the vibrators on and off at random intervals between 2 and 200 ms. The amplitude of the signal was controlled independently for each vibrating element with a potentiometer (10 K-Ohm wheel, 0.5 W). To determine sub-threshold values, the potentiometers were adjusted for each subject until the vibration stimulus was at 90% of the threshold level for each subject. This value was determined by asking the subject whether they felt the vibration on their feet as they stood in the footwear and the experimenter turned the potentiometer dial.

### Procedures

Subjects stood on dual triaxial force plates (AMTI, Watertown, MA) and within three transparent 1.2 m x 1.6 m screens placed 90 cm in front and to the right and left of the force plates. Dense foam (6.5 cm thick) was placed over the forceplates and subjects were instructed to stand quietly. During each 90 sec trial, subjects stood quietly (QS) with eyes closed (EC) or while viewing a visual scene that rotated in continuous upward pitch (PU) at 30 deg/sec (Figure 1, left). Subjects were also tested with these two visual conditions while performing a Fibonacci sequence as a mental calculation (MC) task. Subjects received one trial of each condition and all trials were randomized.

The image on the visual field was composed of white spheres on a black background and was created by two Panasonic PT-D5600U DLP-based projectors that projected a full-color workstation field (1024x768 stereo) at 60 Hertz (Hz) onto each screen (Figure 1, left). Polarized filters placed in front of each projector provided left eye and right eye views of the image on each screen, and passive stereo glasses delivered the correct view to each eye. Three dual processor computers created the imagery projected in the virtual environment and were synchronized via the CAVELib application (MechDyne, Virginia Beach, VA).

### Data collection

Center of pressure (COP) was sampled from the force plates at 200 Hz and normalized to the subject’s initial position. Kinematic data from the head, trunk, lower and upper limbs was collected using a 6-camera infrared Hawk system (Motion Analysis, Santa Rosa, CA) sampled at 120 Hz and low-pass filtered using a zero-lag fourth-order Butterworth filter with a cut-off frequency of 4 Hz. Whole body center of mass (COM) excursions were computed with reference to anthropometric data [16] and normalized to the subject’s initial position at the start of the trial.

### Data analysis

To assess dispersion of the COM and COP, ellipses were fit to the data and the area of the ellipse was calculated. The two main axes of the ellipse were found by calculating the eigenvalues of the covariance matrix between the anterior-posterior (AP) and side-to-side or lateral plane (ML) data [17]. The first eigenvector of the covariance matrix was the direction of the principal axis and the corresponding largest eigenvalue was the variance along this axis. The second eigenvector, which was orthogonal to the first eigenvector, defined the direction of the minor axis and the corresponding eigenvalue was the variance along this axis.

A systematic review of studies investigating the effect of vibration on postural control [18] reported that the majority of studies delivered the vibration stimulus for 30–50 sec. Immersion in a virtual reality environment takes between 8–20 sec for most subjects [13]. Thus, we chose a 30 sec stimulus period for our trials (Figure 1, left). The time taken to reach a steady state whereby the brain could exploit new sensory information following administration or removal of visual and haptic signals is approximately 3 sec [19-21]. Therefore, to avoid transient effects of adding and removing the vibration stimulus, we chose the middle 20 sec of each 30 sec time period for further analysis. Root mean square values (RMS) of the AP and ML COP and COM, and approximate entropy (ApEn) of AP COP and COM excursions were calculated within each time period. ApEn results in a non-dimensional value from 0 to 2 where smaller values of ApEn imply greater probability of repeating sequences (or regularity) of the observations [22,23].

The effects of vibration on postural responses in the presence of visual flow and during the mental calculation task were tested for significance using a 2×2×3 repeated measures analysis of variance (ANOVA) at a 95% confidence level (*p < 0.05)* with a Tukey adjustment for multiple comparisons (version 8.0.1, SAS Institute Inc., Cary, North Carolina). Within-subject factors were visual condition (eyes closed and pitch-up scene), task (standing quietly with and without mental calculation), and comparison of responses across the pre-vibration, vibration and post- vibration periods.

## Results

### Effect of plantar vibration on postural responses

**Figure 2:**
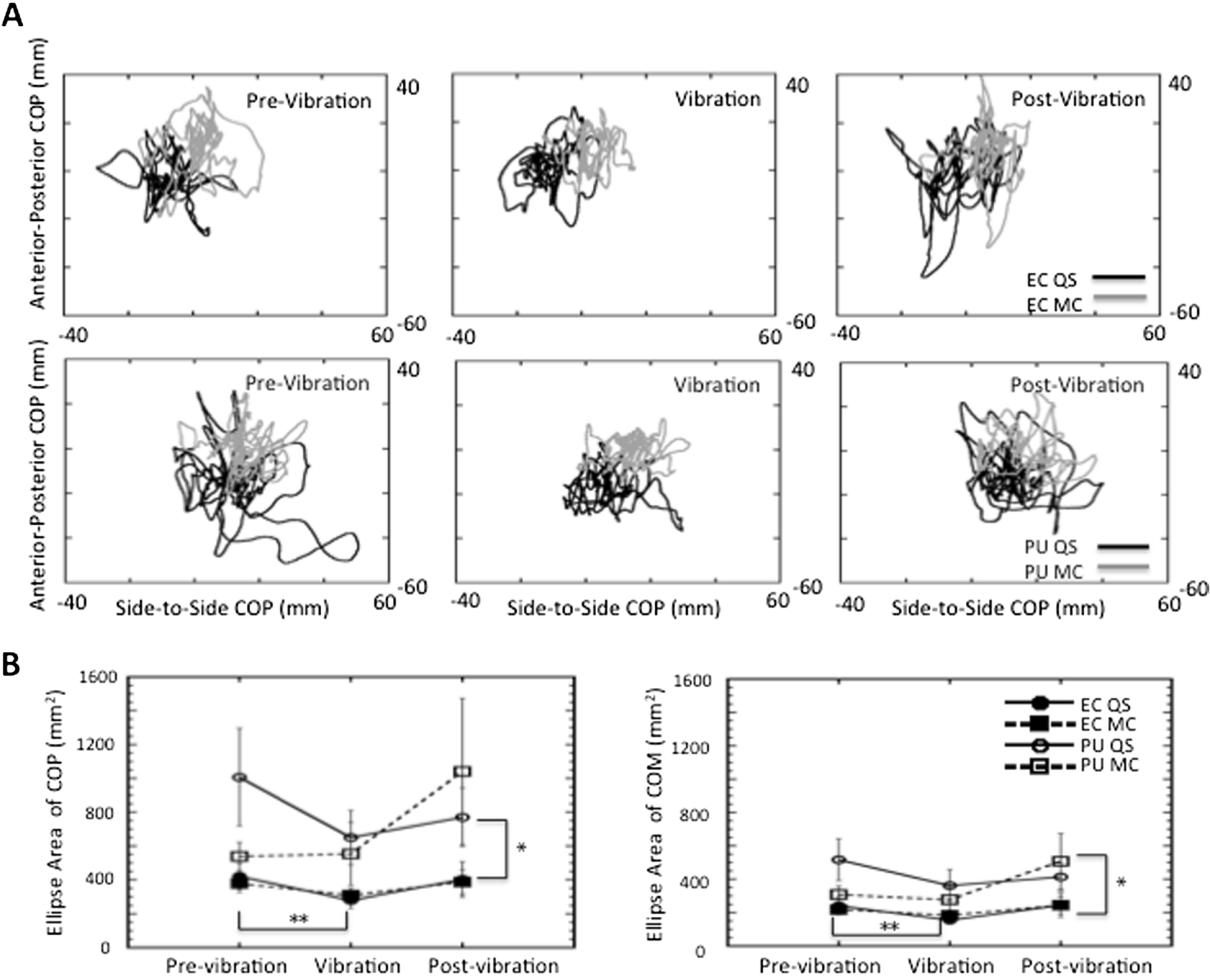
**Excursion of responses over time. A**: AP COP versus ML COP plotted for each time period for a single subject during eyes closed (*top row*) and pitch up rotation of the visual field (*bottom row*) when standing quietly (black line) and when performing mental calculation (grey line). An ellipse was fit to these traces and area was calculated. Ellipses have been omitted for clarity. **B**: Average Area of COP (left) and COM (right) with standard error bars for all experimental conditions.

Post-hoc analyses revealed that the addition of vibrotactile noise to the plantar surface during quiet stance with eyes closed reduced the area of the COM (t(20) = 2.56, p < 0.02) and COP (t(20) = 2.93, p < 0.01) responses which then returned to pre-vibration levels after vibration was removed (Figure 2). The principal axes of the ellipses were determined to be the AP and ML planes. Therefore, subsequent results are given for these two planes.

**Figure 3:**
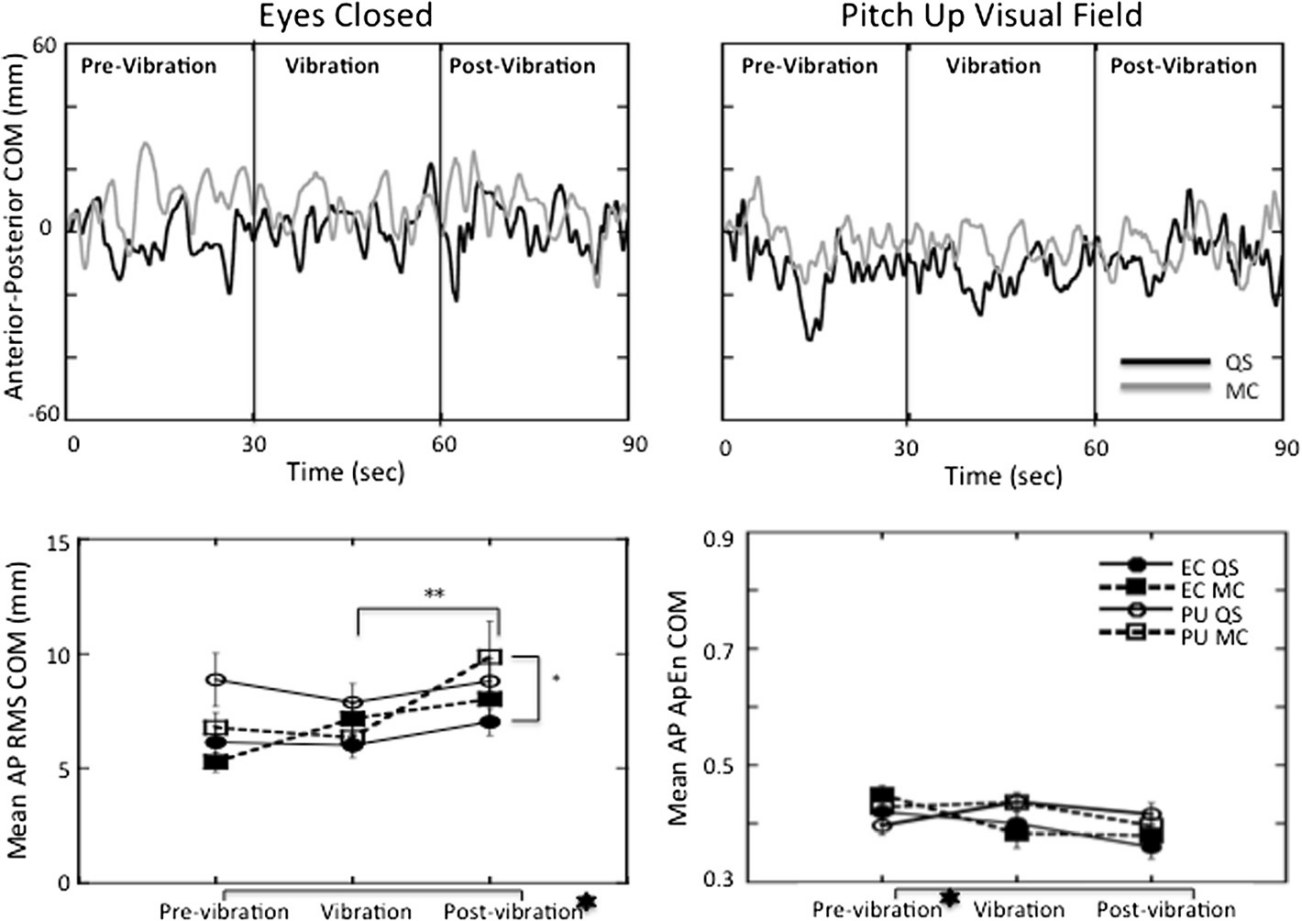
**Anterior-posterior responses of the COM. ***Top*: COM in the AP plane plotted over time for a single subject during the eyes closed (*left*) and the pitch up visual field (*right*) conditions in quiet stance (black line) and when performing mental calculation (grey line). *Bottom:* Average RMS (*left*) and ApEn (*right*) of the COM responses with standard error bars across all subjects in the AP plane. =Significant main effects (*p* < 0.05) between pre-and post-vibration responses; ** = Significant differences (*p* < 0.05) between trial periods; * = Significant differences (*p* < 0.05) between pitch up rotation and eyes closed responses.

**Figure 4:**
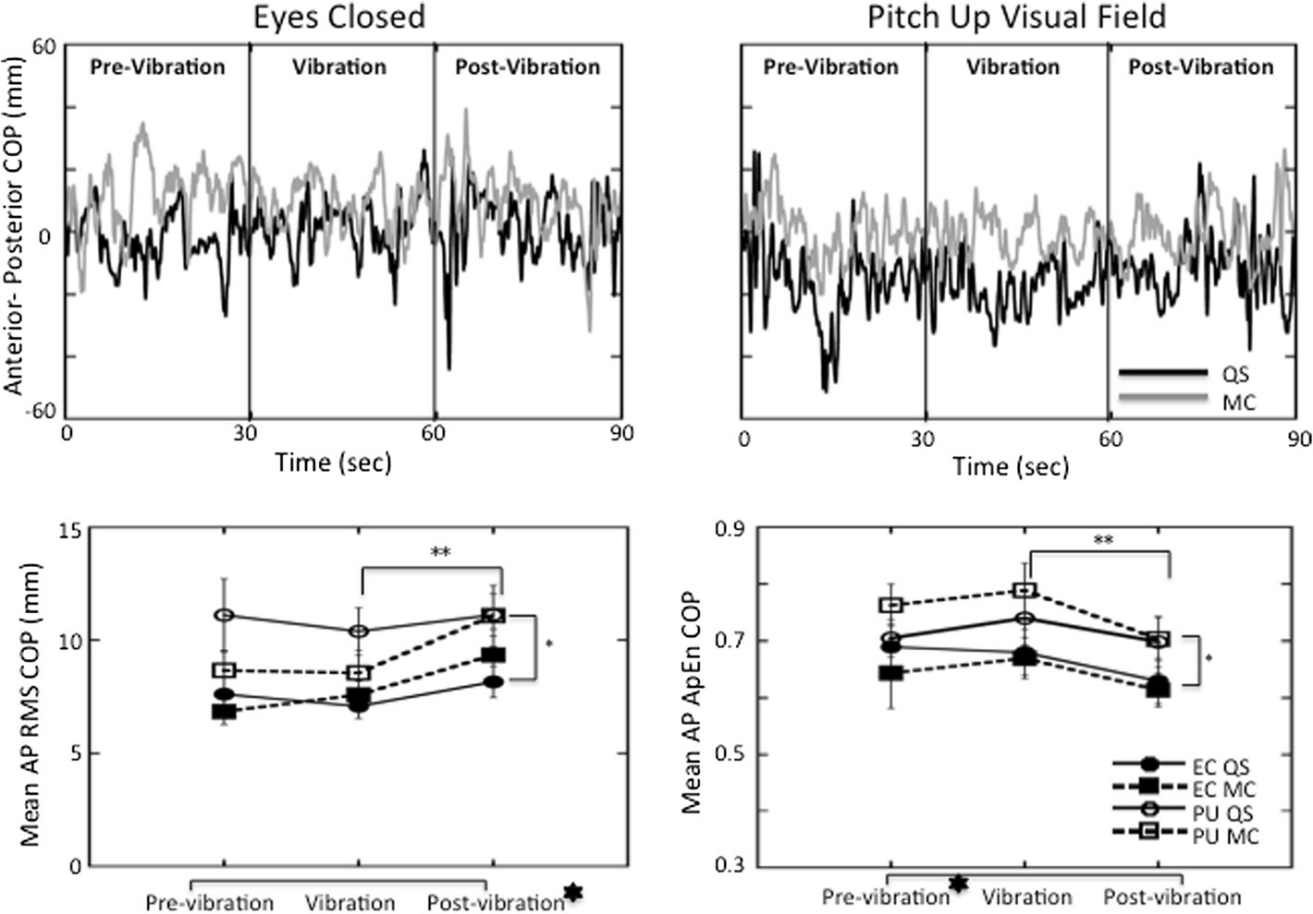
**Anterior-posterior responses of the COP. ***Top*: COP in the AP plane plotted over time for a single subject during the eyes closed (*left*) and the pitch up visual field (*right*) conditions in quiet stance (black line) and when performing mental calculation (grey line). *Bottom:* Average RMS (*left*) and ApEn (*right*) of the COP responses with standard error bars across all subjects in the AP plane. The signs for statistical significance are the same as in Figure 3.

**Figure 5:**
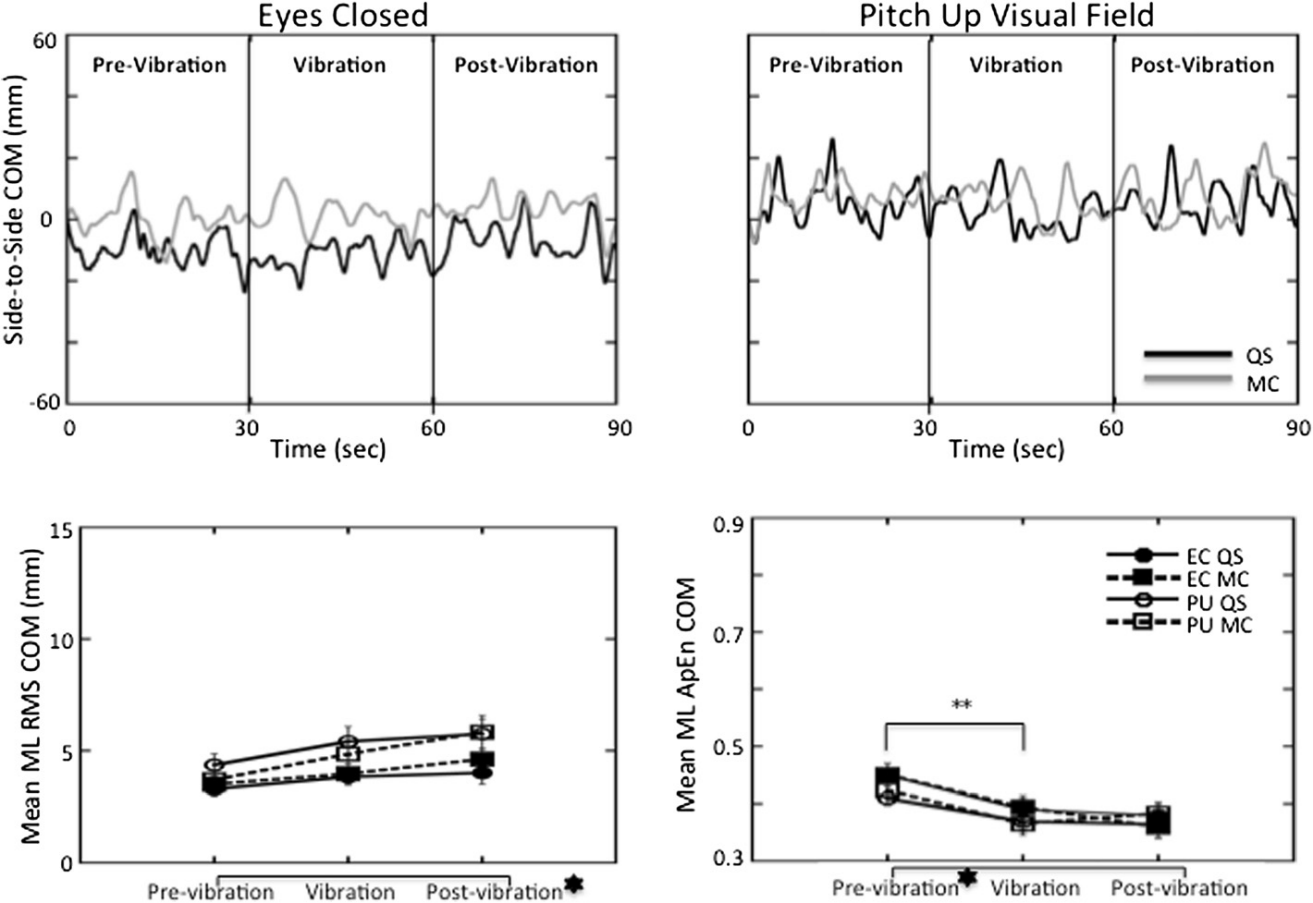
**Lateral plane responses of the COM. ***Top*: COM in the ML plane plotted over time for a single subject during the eyes closed (*left*) and the pitch up visual field (*right*) conditions in quiet stance (black line) and when performing mental calculation (grey line). *Bottom:* Average RMS (*left*) and ApEn (*right*) of the COM responses with standard error bars across all subjects in the ML plane. The signs for statistical significance are the same as in Figure 3.

**Figure 6:**
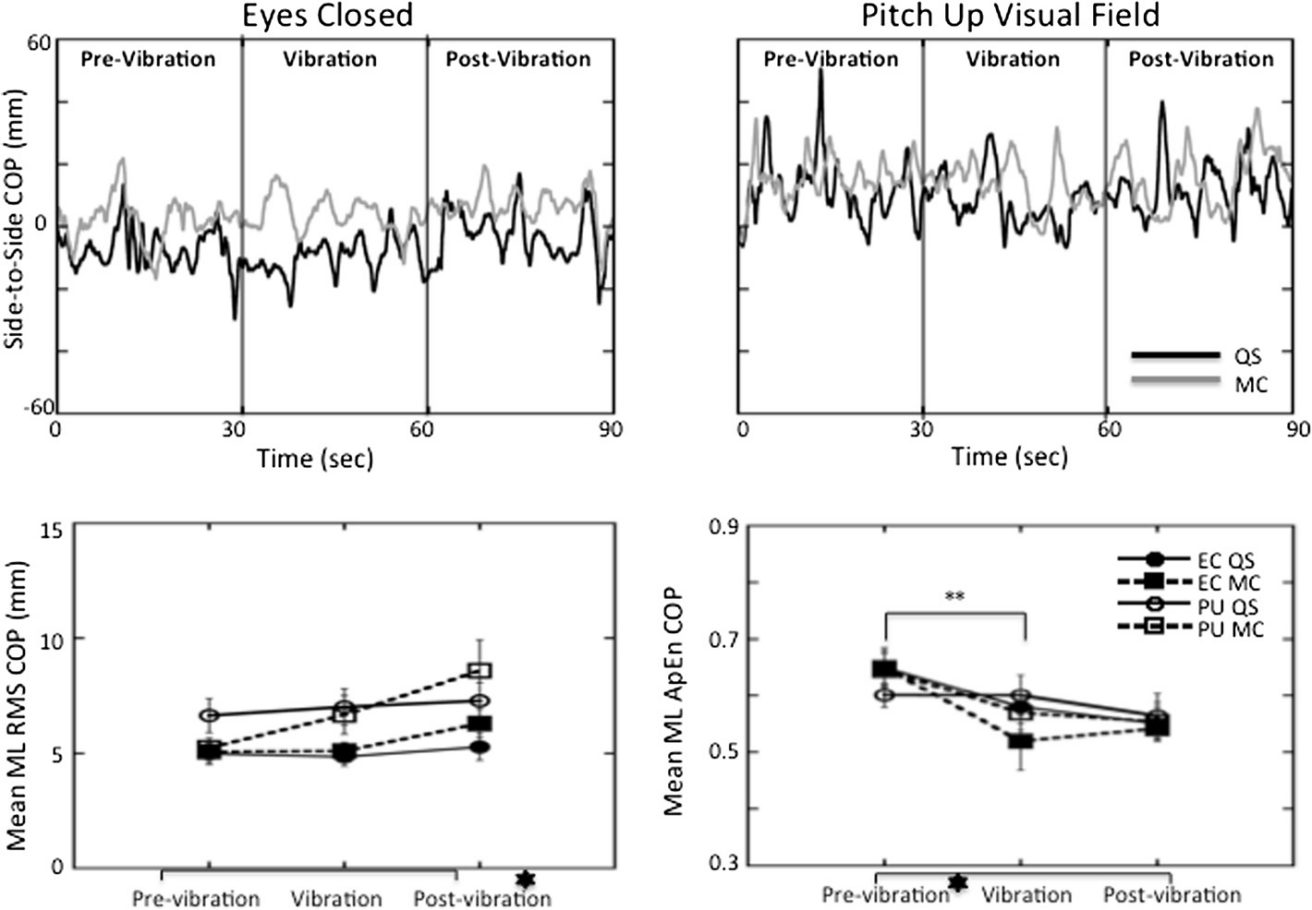
***Top*: Lateral plane responses of the COP.** COP in the ML plane plotted over time for a single subject during the eyes closed (*left*) and the pitch up visual field (*right*) conditions in quiet stance (black line) and when performing mental calculation (grey line). *Bottom:* Average RMS (*left*) and ApEn (*right*) of the COP responses with standard error bars across all subjects in the ML plane. The signs for statistical significance are the same as in Figure 3.

During quiet stance with eyes closed, only the RMS responses of AP COM (t(20) = 2.34, p < 0.03) and AP COP (t(20) = 2.35, p < 0.03) exhibited significant increases between the vibration and post-vibration periods (Figures 3 and 4). A persuasive effect of vibration was also observed in the ApEn values of ML COM (t(20) = 2.68, p < 0.01) and COP (t(20) = 2.36, p < 0.03) which decreased significantly between the pre-vibration and vibration periods during quiet stance with eyes closed (Figures 5 and 6) suggesting greater regularity in the response with the addition of the vibration stimulus.

### Performing a mental calculation task

Performing the mental calculation task produced greater amplitudes and significantly greater RMS values (F(1,20) = 56.9 p < 0.001) in AP COP responses than during quiet stance (Figure 4). ApEn of the AP COP values were significantly lower (F(1,20) = 325 p < 0.001) across the period of the trial with mental calculation than during quiet stance (Figure 4).

Significant interactions emerged when mental calculation was combined with vibration. A larger increase was observed between periods of the trial in both AP COP (F(2,40) = 6.19 p < 0.005) and AP COM (F(2,40) = 4.55 p < 0.02) RMS responses (Figures 3 and 4). A significant effect (F(2,40) = 4.07 p < 0.025) of mental calculation was also observed in the AP COP ApEn responses that increased between the pre-vibration and vibration periods, and then decreased in the post-vibration period with mental calculation (Figure 4).

### Viewing a moving visual field

A moving visual field significantly increased magnitude of the AP COM (F(1,20) = 10.9 p < 0.004) and COP (F(1,20) = 25.7 p < 0.001) responses (Figures 3 and 4), and this effect was reflected in increased ApEn values of COM (F(2,40) = 5.70 p < 0.006) and COP (F(1,20) = 427 p < 0.001) in the AP plane (Figures 3 and 4). Compared to the eyes closed condition, visual field motion also significantly increased the area of the COM response across all periods of the trial (Figure 2) during both quiet stance (pre-vibration = t(20) = 3.16, p < 0.05; vibration = t(20) = 2.70, p < 0.01; post-vibration = t(20) = 3.87, p < 0.001) and mental calculation (pre-vibration = t(20) = 2.42, p < 0.025; vibration = t(20) = 3.88, p < 0.001; post-vibration = t(20) = 2.07, p < 0.05).

COP planar motion (Figure 6) presents a comprehensive picture of the effect of the additional task demands on the postural sway. Although responses were generally larger in the AP plane (compare Figures 4 and 6), AP RMS responses significantly increased (F(1,20) = 58.0 p < 0.001) when standing in the pitching visual field while performing a mental calculation task compared to eyes closed responses in the pre-vibration (t(20) = 6.06 p < 0.0001), vibration (t(20) = 5.70 p < 0.0001), and post-vibration periods (t(20) = 5.82 p < 0.0001). Even with a steep decrease in area of COP when vibration was added (Figure 2), COP area was still significantly greater during quiet stance in a moving visual field than with eyes closed in the pre-vibration (t(20) = 2.56, p < 0.0186), vibration (t(20) = 3.02, p < 0.0067), and post-vibration (t(20) = 4.05, p < 0.0006) periods. Higher ApEn values of AP COP (Figure 4) in the pre-vibration (t(20) = 20.56 p < 0.001), vibration (t(20) = 13.51 p < 0.001), and post-vibration (t(20) = 15.72 p < 0.001) periods when mental calculation was performed in a moving visual field confirm an increased complexity in responses to the combined tasks compared to eyes closed conditions.

## Discussion

Previous data suggest that adding a noisy signal to an unstable system will improve postural stability in several clinical populations [4,6,7,24,25]. But to produce functional stability, this signal needs to be effective during complex tasking requiring the simultaneous processing of multiple task demands. In this study, we explored whether a stochastic vibrotactile signal effectively modified standing balance when delivered during a task that also presented visual and cognitive demands. Our results indicate that the effect of sub-threshold vibratory noise on postural behavior was modified when combined with other sensory and cognitive demands that generated increased postural instability. Specifically, we found that there were significant interactions between the responses to vibration and responses to a moving visual field or an additional cognitive load. In addition, we were able to show that the measure of approximate entropy reflected increased task complexity.

Based on prior reports [6,7,25], our expectation was to find reduced postural sway during the application of sub-threshold vibration and this was indeed the case. Unexpectedly, the size of the postural responses rebounded when vibration was removed. Responses in the post-vibration period exhibited their greatest increase during the eyes closed, quiet stance condition, suggesting that vibration had its strongest effect on reducing postural sway when there were no additional task demands. We might infer that a dynamic sensory re-weighting of the sensory inputs occurred as task conditions changed [26-28], and enhanced tactile sensitivity during application of stochastic resonance [6,7,24] was downgraded or upgraded depending on the existence of other task demands.

Maintaining balance with an additional cognitive load or within a rotating visual field increased the size of the postural kinematics [3,13], and directionally modified the postural responses so that there was greater motion in the anterior-posterior plane. When vibration was removed, lateral plane sway responses also increased in size. This suggests that the presence of vibrotactile input reweighted the system to enhance tactile sensitivity and reduce sensitivity to the destabilizing effects of the calculation task and visual field motion. This could have a significant clinical implication as increased sway oscillations in the lateral plane have been shown to positively correlate with a history of falls in older adults [29,30].

We employed approximate entropy (ApEn) as a non-linear measure of the variability in the temporal structure of sway because of increasing evidence of non-linear control mechanisms for postural control [31,32]. It is not clear whether there is an optimal state of variability for functional movement, but it has been shown that biological systems that are either overly rigid or noisy are also unstable [33].

We expected that the lowest ApEn values (i.e., greatest response regularity) would occur during quiet stance with eyes closed [34], and that ApEn values would increase with the addition of task demands. Response complexity did increase with the addition of vibration, particularly when combined with motion in the visual field. Response complexity tended to decrease (lower ApEN values) when vibration was removed, but less so in the lateral plane during the mental calculation task. Thus, increased sway did not necessarily reflect degraded balance [35,36] and increased variability was not indicative of ineffective postural control [33,36].

Our results support the concept of a continuum of stability that was influenced by the confluence of particular task requirements [37]. Of course, there are some limitations to this study including the number and frequency of the vibrotactile sensors, their placement on the foot, and the time intervals selected for the trial periods. It is possible that if we delivered vibration for a longer period of time, we might have reduced the after-effects and sustained the effect of the vibration. Clearly this needs to be investigated further if we are to determine a clinical impact of this approach.

## Conclusions

There is a close relationship between increased sway and impaired lower limb sensation due to an inability to detect changes in upright position [38]. Our results suggest that the impact of other destabilizing signals is modulated when combined with vibrotactile stimulation. The strong aftereffects of the vibration stimulus suggest that the system has adapted to the sensory array even in the short time period tested here [39]. Although any conclusions are constrained by the population selected for study and the limited timeframe that the stimulus was applied, the results imply that application of vibrotactile stimulation has the potential for diminishing sway magnitudes while increasing the potential for response variability, thereby presenting a non-invasive method of reducing the potential for falls [40].

## Competing interests

The authors declare that they have no competing interests.

## Authors’ contributions

All authors have made substantial contributions to conception, design, acquisition of data, or analysis and interpretation of data. EK conceived the study and supervised data analysis and interpretation of data and was primary author on this manuscript. JS contributed to data collection, data analysis, and drafting the manuscript. LLD contributed to design, acquisition of data, analysis and interpretation of data and approved the manuscript. KD participated in data analysis and interpretation and approved the manuscript. All authors have given final approval of the version to be published.
